# Report of a Special Committee of the Medical Society of the District of Columbia, upon the Claims of Homoeopaths and Other Irregular Practitioners for Professional Recognition in the Medical Service of the United States Government, and the Charges Brought by the Homoeopaths against the United States Commissioners of Army and Navy Pensions

**Published:** 1871-04

**Authors:** 


### Miscellaneous.

### Report of a Special Committee of the Medical Society of the District of Columbia, upon the Claims of Homoeopaths and other Irregular Practitioners for Professional Recognition in the Medical Service of the United States Government, and the Charges brought by the Homoeopaths against the United States Commissioners of Army and Navy Pensions.

The committee beg leave to report that the present antagonistic attitude of the homœopathic practioners towards the United States Commissioner of Pensions had its origin in the well-directed labors of the latter functionary to simplify and render more uniform the medical action of that bureau.

Some years ago, and up to the commencement of the civil war, the Pension Bureau was of small dimensions, and employed a limited staff to perform the duties, but when the close of the war found many thousands ot men distributed over the United States, applicants for pension on account of wounds and disease incurred in the line of military or naval service, and which more or less incapacitated them for earning their own livelihood or sustainiag their families, the functions of the Pension Bureau became extended and onerous, involving the employment of a large medical staff demanding not only good general education, but special aptitude for diagnosis and a thorough acquaintance with the nature and consequences of that class of injuries and diseases incidental to camp and active field service; that the medical men employed by the Pension Bureau have not always possessed the qualities stated is simply a statement of fact, nor could the mass well be otherwise, since they were not appointed by examination into their qualifications nor for any special fitness, but simply chosen from the locality on the recommendation of some one who furnished, when required, the name of one or more practitioners of his locality. Political influence often pressed these medical men upon the bureau, and their influence often occasioned the selection not of the fittest man, but of him most influential in local politics, and thus, from one cause or other, the medical qualifications of pension surgeons were wholly lost sight of; and hence it was that when Commissioner Van Aernam entered on the duties of the bureau he found all degrees of medical standing, all classes of practitioners, regular and irregular, on the rolls. There were eclectics and Thompsonians, Indian doctors, herbalists, hydropaths, homoeopaths, and abortionists, according to their own written statement.

At the time stated there were 1,350 surgeons on the roll, of whom 1,312 were regular practitioners; of the residual 38 one-half, or 19, were homoeopaths, and of this latter number 17 claimed to have graduated in colleges of regular medicine; the other 19 were filled up by the *soi-disant* physicians alluded to.

Thus, by the above-stated method of appointment, this heterogeneous mass of practitioners became attached to the bureau under former commissioners, who, not being medical men, could not, and cannot be expected to appreciate the inefficiency and weakness of a medical board so constituted; nor was it rendered so apparent how impossible it was to unify the action of the officer with such a discordant force until very lately, when under the present commissioner the mode of examination of pensioners throughout the whole United States was altered, so that, instead of the pensioner applying to a single pension surgeon, and being examined by him and a report forwarded to the bureau at Washington by a single surgeon, boards of examination were ordered to convene in the localities, before whom the pensioner appeared, when a joint examination and joint report was made out and forwarded.

By altered arrangement Commissioner Van Aernam hoped to perform the duties of his office intelligibly, and to the best advantage of the government and the true interest of the soldier.

As soon as these boards commenced to be convened, it became apparent to the bureau that the efficiency and harmony essential could not be attained, since, according to the established rule of medical ethics, the regular physician refused to attend or consult on the same board with the homoeopath, and to avoid obstructing the business of the office, attain unity of action and justice to the pensioner, one or other physician must give way.

In such cases the homoeopath does not feel called on to recede, since his code of ethics allows him, practically to consult with all physicans. But the ethics of the regular require him to decline or withdraw from such association, and, as the majority of all these boards are made up of regular physicians, such action would break up every board, and deprive the Pension Bureau of its most important and experienced medical advice. Now, as the ratio of this class of practitioners is to the number of regular physicians on the roll of examining surgeons as *one* to sixty one, admitting, for argument's sake, although it is by no means true, both classes to be equally well educated and capable of serving the government, it is obvious that the simplest mode of removing the difficulty and obviating harmony, was to eliminate this one from every sixty-one; and this is what Commissioner Van Aernam did. The assumed rights of one physician should not become a stumbling-block when so preponderating a number of the other class existed.

But it is idle to assert for one moment, or admit, that the regular physician and the homoeopath is of equal benefit to the office, for it is mainly surgical advice which is needed, and a full acquaintance with that portion of military surgery which is occupied with the treatment and results of gunshot injuries. Such information is only possessed by those who were surgeons in the late war, or who have served or held the office of surgeon in city surgical hospitals; as these latter are a very small class, they need not be considered; and it may be stated, therefore, that not to secure the services of the volunteer medical men of the war would have been reprehensible, and the Commissioner would have been open to severe animadversion for malfeasance had he neglected to secure such counsel, even at the loss of irregular practitioners equally well-educated on other points. Commissioner Van Aernam had, then, no alternative but to drop the irregular in order to secure harmony and efficiency in the action of these boards. Besides, it was most natural for the Commissioner to select from that class of men who, and *who alone* were eligible to and did service in the army and navy. Possessed with this conviction, and solely in the interest and regular discharge of the duties of his office, he addressed a circular letter, dated May 25, 1870, to each examining surgeon, headed "Personal Report of Examing Surgeon," and containg certain queries. The blank is as follows:

PERSONAL REPORT OF EXAMINING SURGEON.

Dated, ————————, 187 .

Name in full,————; residence,————; county of ————; State of ————; graduated at ————; diploma dated ————; nature of military or naval service (if any) ————; *under what system of medicine do you *practice?*

*This question can be answered in one word, thus, "allopathic," "homœopathic," "hydropathic," "eclectic," &c., &c., as the case may be.

The replies contained in the personal report abundantly sustained his judgment and furnished the means of ascertaining the experienced, the competent, and the irregular practitioners. The latter immediately after received a letter requesting them to withdraw their names from the lists of examining surgeons, and from that date the office of Examining Surgeon for the Pension Office has been wholly in the hands of regular medicine.

The elimination, however, was not confined to this class only, for it proceeded to the extent of removing over five hundred additional examining surgeons who were not deemed competent, although regular in their mode of practice and medical education. Among the parties addressed was Dr. Stillman Spooner, of Oneida, New York, who, acknowledging his homoeopathic practice, also was requested to resign. Shortly after he addressed a letter to the commissioner protesting against his action, calling it a proscription of opinion, and endeavoring to excite political feeling and introduce it as a reason why homoeopaths should share these offices.

The further history of this matter may be stated in a few words. Homoeopaths in one or more of the states agitated this "grievance," as they termed it, by meetings and by inflammatory articles in the newpapers, fanning political party flames, exciting a false public sentiment, and indulging in arguments and statements wholly at variance with truth. The Homoeopathic State Medical Society of New York, at their meeting, held in the city of Albany, passed resolutions denunciatory of the commissioner, and demanding his removal for this act of displacing irregular practitioners, and a deputation during last month waited on the President and Secretary of the Interior calling for the quick removal of the commissioner; and thus the matter stands.

From various sources, public and private, official and personal, the commissioner has received an unanimous endorsement of his proceedings from the profession; and your committee, in reviewing the whole affair, are of opinion that the action of the commissioner, while it was conducive to the best interests of the bureau over which he presides, by availing himself of medical advice, whenever practicable, only from physicians who, having served in the late civil war, are certainly the most competent to form opinions on wounds and disease of military life, that action was also in accordance with thev iews of the whole medical profession of this country, with whom homoeopaths and other irregular practitioners have no professional status.

Your committee would call attention to the arrogance and untruths contained in the statements of the irregulars in this contest to secure place and position under the goverment. The homoeopathists have the temerity to say they are 10,000 in the United States, but a careful collection of the actual returns show that the whole number in our country do not exceed 3,000. Dr. S. Spooner says, in his published letter: "In the village of Oneida, my place of residence, there are eight physicians, four belonging to each school. We recognize each other as physicians on equal terms." It sounds rather strangely to our ears to hear that four regular physicians, the whole hope of a village, can be so forgetful of their ethical vows as to consult promiscuously with irregulars, and we are relieved to learn, from a communication dated from the same Oneida, Dr. S. Spooner's village, in which he is accused of conduct unbecoming a physician; and that, "by homoeopathic physicians in this vicinity, he is considered irregular in his practice, and is seldom, if ever, called into consultation." The writer of the same communication goes into the detail of medical acts of Dr. S. Spooner, which, if previously known to the Pension Office, would doubtless have led to his instant dismissal from his position as pension surgeon; and this is the man whom the homoeopaths deem worthy to put forward as a case of oppression and proscription of opinion.

Having now shown that, for the sake of the due performance of the medical duties of the bureau, it was essential that only regular physicians should be selected, and that the number of class of irregular practitioners is so small that they ought not to be considered, in view of the overwhelming majority of opinion on the side of the regulars, your committee desire to place before you some further considerations. The homoeopaths demand their right to be appointed to these positions on account of their education, their number, and influence, and assert that they are kept unjustly out of positions in the Army and Navy Medical Staff solely from the jealousy of legitimate medicine, and that, although few in number they are entitled to representation as far as their numbers go. I

is no doubt true that numbers do not give the right, and that majorities are apt to be exclusive; but this only true when the numbers represent a heterogeneous assembly. On matters strictly professional, whatever be the profession, the majority are likely, nay, indeed, almost certain, of being on the right side. and this majority constitutes the voice and authority of the profession, regulates and controls the whole body, and the dictum of that majority is accepted as the professional axiom. The courts of law regulate the practice of the bar, and no attorney dare act or practice in opposition to the rules of court. In Episcopal religions the Bishops give the formula, and the minister who disputes or practically differs is disrobed; and how would the public treat any complaint of injustice, oppression, and illiberality against these governing bodies? The only governing body in medicine in this country is the American Medical Association, the representative organ of the whole regular profession.

Medical men, and this body has declared in its code of ethics that consultations are only to be held with regular practitioners, and that "no one can be considered as a regular practitioner or a " fit associate in consultation whose practice is based on an exclu- "sive dogma to the rejection of the accumulated experience of the "profession and of the aids actually furnished by anatomy, physi- "ology, pathology, and organic chemistry," (Code of Med. Ethics, Art. 4, §1,) and any surrender of this rule should be considered a step backward in the profession.

The power of our profession over the entire public rests not on jealousy and illiberality nor on numbers, but on a consciousness in that public that we represent the progress of medicine from apostolic times in continuous succession, from which all smaller sects of practitioners are offshoots, fostered by ambition, vanity, and continued by obliquity of intellect or sordid self-interest; that regular medicine rests not on the dogma of a single teacher, which may be modified to suit the knowledge of the present day, but upon an humble, faithful and world-wide observation of the laws of nature, verified and proved and made manifest over and over until he that runs may read, and changing, altering, and improving its practice in accordance with the lights of all the sciences. If this be so, and the experience not only of this country but of Europe and the whole civilized world proves it, since everywhere almost without exception regular medicine is entrusted with important governmental medical offices and support; then is the reason evident that regular medicine only should be called in to serve the government, and that homoeopathy or other irregular sects in medicine, no matter how numerous or influential, politically or otherwise, it may be, should not be represented in such situations.

Your committee, in conclusion, recommend the adoption of the following preamble and resolutions:

WHEREAS, the large majority of the present examining surgeons of the Pension Bureau have served in the medical corps of the volunteer forces during the late war; and whereas, none but regular physicians were admitted into that corps of the regular army and navy, and therefore none but regular physicians are provided with the medical experience requisite on examining boards: therefore,

*Resolved*, That this Society deems the action of the Hon. Commissioner of Pensions, in excluding irregular practitioners from the Medical Examining Board under that bureau as made in the best interest of the public service, thereby leading to uniformity of action, increasing the efficiency of the bureau, and affording to the pensioner the benefit of the most skilled advice; and it is earnestly hoped that the government will not in this instance disregard the deliberate and expressed conviction of the whole legitimate medical profession of this country by appointing to medical position or office a class of men whose practice is not based on experience and observation, the only true groundwork of medical progress, but upon arbitrary dicta, not verified after nearly a century of trial, and which are wholly opposed to the ordinary exposition of the natural laws of physical science.

*Resolved*, That a copy of the foregoing resolutions be respectfully forwarded to the President of the United States and the Secretary of the Interior.

(Signed,) THOS. ANTISELL, M. D., Chairman.
THOMAS MILLE, M. D.,
LOUIS MACKALL, JR., M. D.
J. M. TONER, M. D.

